# How do I evaluate myself? The importance of examining overevaluation of muscularity in risk for eating disorder symptoms

**DOI:** 10.1186/s40337-025-01419-3

**Published:** 2025-10-23

**Authors:** Chloe White, Michael Maraun, Shannon Zaitsoff

**Affiliations:** Department of Psychology, Simon Fraser University, 8888 University Drive, Burnaby, BC V5A 0A9 Canada

## Abstract

**Background:**

The extent to which individuals view, think and feel about their shape and weight in relation to their self-esteem is understood as a risk factor for eating disorders. However, muscularity has yet to be examined as an appearance category that individuals may base their self-esteem on. Thus, this study examined whether evaluating oneself based on muscularity (overevaluation of muscularity) may be relevant to men and women’s self-esteem and whether this form of self-evaluation may relate to eating disorder symptoms most prominently in men, who frequently present with muscularity concerns.

**Method:**

Young adults (*N* = 290; 50.3% cisgender women) were recruited from a Canadian university and completed a modified version of the Shape and Weight Based Self-Esteem Questionnaire and a measure of eating disorder symptoms.

**Results:**

Men endorsed greater overevaluation of muscularity than women, although women endorsed greater overevaluation of shape and weight than men. Despite differences in the forms of appearance on which men and women based their self-esteem, multi-group structural equation models demonstrated that there were no differences in the associations between overevaluation of shape-, weight-, and muscularity and eating disorder symptoms across men and women. However, overevaluation of shape-, weight-, and muscularity were associated with distinct eating disorder symptoms.

**Conclusions:**

Altogether, results provide nuanced information regarding the importance of assessing self-evaluation based on muscularity, alongside shape- and weight, as increased self-evaluation based on these appearance domains may confer risk for eating disorder symptoms.

**Supplementary Information:**

The online version contains supplementary material available at 10.1186/s40337-025-01419-3.

## Abstract (plain language summary)

Basing self-esteem on appearance, such as shape or weight, is shown to be a risk factor for increased eating disorder symptoms. However, basing self-esteem on the appearance of one’s muscles may also be an important appearance category; especially for men who frequently endorse muscularity-related concerns. Despite this, no research has sought to examine whether men or women base their self-esteem on muscularity and, further, whether this may relate to eating disorder symptoms. Thus, this study examined 1) whether men and women base their self-esteem on shape, weight, and muscularity, and 2) whether basing self-esteem on these forms of appearance related to eating disorder symptoms differently for men and women. Results showed that while men were more likely than women to endorse self-esteem based on muscularity, basing self-esteem on any appearance category was related to eating disorder symptoms in both men and women. These results underscore the importance of inquiring about muscularity-related concerns for men and women, especially in the context of understanding risk for eating disorder symptoms.

**Supplementary Information:**

The online version contains supplementary material available at 10.1186/s40337-025-01419-3.

## Introduction

Low global self-esteem has been identified as a core risk factor related to eating disorders (ED) (see for review, Colmsee et al. [8]. Yet, more specifically than global self-esteem, the extent to which individuals base their self-esteem on their appearance (overevaluation of appearance) is consistently related to the development and maintenance of ED symptoms [1, 30, 40]. The form of the relationship between overevaluation of appearance and ED symptoms may differ for men and women, in consequence of differences between the body ideals most relevant to men versus women. For instance, women’s body ideals draw largely on the qualities of thinness, leanness, and tone. Thus, shape (i.e. an individual’s current shape, the way their clothes fit) and weight (e.g., an individual’s actual current weight) may play a greater role in determining appearance-based aspects of self-esteem for women than for men. Alternatively, body ideals for men overwhelmingly feature the pairing of muscular hypertrophy and leanness. Thus, while muscularity-oriented goals may encompass weight and shape-related concerns, such as weight loss, muscularity-oriented goals which focus on enhancing the size of muscles may serve as a primary motivation for ED behaviours in men [22, 23, 29]. Accordingly, men are more likely to engage in muscle building and excessive exercise, while women are more likely to engage in restricting and purging behaviours [16]. However, although muscularity concerns predict ED behaviours in men, no research has sought to examine whether men and women over evaluate themselves based on different aspects of appearance, such as the size and tone of their muscles, and how these differences relate to ED behaviours. Thus, we sought to elucidate the web of relationships extant between ED symptoms and the self-assessed impact of basing self-esteem on shape, weight, or muscularity for men and women.

The Transdiagnostic Model of Eating Disorders suggests that common maintenance mechanisms operate across ED diagnostic categories [12–14]. Specifically, the model emphasizes overevaluation of appearance and the role of a dysfunctional system of self-evaluation as central to ED pathology. Research supports this model, showing that overevaluation of shape and weight relates to decreased global self-esteem in clinical and community samples [20, 42, 43] and that the greater portion of total self-esteem that shape and weight occupy, the greater likelihood of experiencing disordered eating symptoms [20, 21]. Importantly, the extent to which individual’s over emphasize the importance of shape and weight in their self-esteem is also suggested as an indicator of ED prognosis and severity. For instance, research shows that overevaluation of shape and weight distinguishes individuals with more severe EDs from those with less ED symptoms [36]. Further, studies show that those who exhibit severe overevaluation of shape and weight benefit less from ED treatment than those with moderate overevaluation [35] and are more likely to relapse in their ED symptoms following treatment [25]. However, overevaluation of shape and weight does not solely occur for those already diagnosed with ED’s; in fact, prominence of overevaluation of shape and weight is on the rise in the general population [39] and has been shown to be a risk factor for ED symptoms and their comorbidities.

Much research has examined the association between overevaluation of shape and weight and ED symptoms in females [6, 10, 13, 20]; however, little research has sought to clarify how overevalution of appearance may relate to ED symptoms in men. Further, research has primarily examined two components of appearance when examining overevaluation: shape and weight. However, overevaluation of muscularity may be particularly important to assess alongside shape and weight in the context of ED symptoms in men. Shape broadly relates to an emphasis on curvature of one’s waist or hips, flat stomach, and the experience of how clothes fit, yet muscularity is specifically oriented to the tone, size, and curves of muscles. Muscularity concerns relate to ED symptoms in men [24] and are more prevalent in men than women [26]. Yet, recent research also highlights the relevance of muscularity concerns for women, with emerging research suggesting the importance of a ‘fit-ideal’ encompassing both thinness and muscularity for women [5, 11]. Of importance, muscularity concerns and goals qualitatively differ across genders, with men often seeking to enhance muscle size nearly exponentially and women seeking to increase muscularity tone and size to be “just right” [3, 32, 33]. Thus, muscularity may be a relevant and distinct appearance domain that men, and some women, base their self-esteem; yet no research has examined the relevance of muscularity alongside shape when examining overevaluation and ED symptoms.

Given the paucity of research examining the association between overevaluation of shape, weight and muscularity and ED symptoms in men, this study had three aims: (1) examine overevaluation of shape, weight, and muscularity in men and women and (2) examine whether associations between overevaluation of shape, weight and muscularity and ED symptoms (e.g., binge eating, purging, restriction, excessive exercise, muscle building) differ in men and women. We hypothesized that:

(a) Women will base more of their self-esteem on shape and weight than men, and (b) men will base more of their self-esteem on muscularity than women.
Gender will moderate associations between overevaluation of shape, weight, and muscularity and ED symptoms. Specifically: (a) For men, in comparison to women, significant associations will be between overevaluation of shape and muscularity and excessive exercise and muscle building; (b) In women, in comparison to men, significant associations will be between overevaluation of weight and shape and restriction and purging.

## Method

### Participants and procedures

Participants were undergraduate students recruited from the psychology participant pool at a large Canadian university in 2023. Eligible participants were between the ages of 18–25. Participants completed a battery of questionnaires including demographic information and questionnaires assessing self-esteem, body image, muscle dysmorphia, and ED symptoms in exchange for course credit. After providing informed consent, participants completed online questionnaires in the same order. On the final page of the survey, participants were directed to an external link (https://gleaming-brioche-43ce26.netlify.app) where they completed the final measures pie chart (i.e., the Shape and Weight Based Self-Esteem Inventory). Three attention-check questions were included for respondents to correctly respond prior to advancing. All components of the study were approved by the Simon Fraser University Research Ethics Board (REB #30001870).

Of the 346 participants who completed the study, 9 participants were excluded for being outside of the accepted age range, 14 were excluded for failing both attention checks, and 29 participants were excluded due to failure to complete the Shape and Weight Based Self-Esteem Inventory. Thus, the final sample for analyses included 290 participants (50.3% cisgender women, 49.7% cisgender men). Participants ranged in age from 18 to 25 years (*M =* 19.21, *SD =* 1.55). Most participants identified as White (29.0%), South Asian (21.7%), and East Asian (23.1%). Detailed demographic information is presented in Table 1.

**Table 1:** Demographic information

Characteristic	*n* (%)
Gender	
Female	146 (50.3%)
Male	144 (49.7%)
Sexual orientation	
Heterosexual	216 (74.5%)
Bisexual/Pansexual	32 (11.0%)
Pansexual	8 (2.8%)
Queer	3 (1.0%)
Gay	4 (1.4%)
Lesbian	1 (0.3%)
Questioning or unsure	15 (5.2%)
Other or Prefer Not to Answer	6 (2.0%)
Ethnicity	
White	84 (29.0%)
Black	4 (1.4%)
Hispanic	9 (3.1%)
South Asian	63 (21.7%)
East Asian	67 (23.1%)
Southeast Asian	29 (10.0%)
Middle Eastern	10 (3.4%)
Multiracial	16 (5.5%)
Indigenous	3 (1.0%)
Prefer Not to Answer	5 (1.7%)
Parental Income	
Unemployed or disabled	2 (0.7%)
Under $50,000	35 (12.1%)
$51,000-100,000	63 (21.7%)
$101,000-200,000	69 (23.8%)
Over $200,000	22 (7.6%)
Unknown or prefer not to answer	99 (34.1%)

## Materials

### The shape and weight based self-esteem inventory (SAWBS; Geller et al. [19])

Participants completed a version of the SAWBS that was adapted for administration online (versus paper and pencil) and included muscularity as one of the domains. The SAWBS is an interactive measure wherein participants view a pie that represents their entire self-esteem and a list of domains that they can use to indicate the extent that different components make-up their self-esteem pie. The domains include intimate or romantic relationships, body shape, body weight, muscularity, competence at school/work, personality, friendships, face, personal development, competence at activities other than school/work, and other (individuals write in an attribute not covered in the list). Importantly, individuals were given example definitions of shape as being their actual current shape or the way their clothes fit, weight as being their actual current weight, and muscularity as being their actual muscle size and tone. Individuals select whichever components (including a write in option if desired) are important to how they have felt about themselves over the past four weeks and rank the components in order of how much their opinion of themselves is based on each. Respondents then indicated the extent that each attribute made up their total percentage of self-esteem, which automatically appeared as a portion their pie.

The computed score represented the extent to which participants overevaluated their shape, weight and/or muscularity. This was derived by the percentage of space occupied by shape, weight, or muscularity domains in the pie. The score reflects the exact percentage of the pie that each domain occupies out of 100; thus, it represents the degree that individuals’ self-esteem is based on shape, weight, and/or muscularity regardless of individuals contentment with each of these domains. The SAWBS has demonstrated excellent convergent validity with existing measures of self-esteem and excellent 1-week test-retest reliability in a sample of young women (*r* = .81; Geller et al. [20]. Importantly, this is the first study to date to examine the SAWBS in a sample of men.

### Eating pathology symptoms inventory (EPSI; Forbush et al. [17])

The EPSI is a 45-item questionnaire assessing disordered eating symptoms over the last 4 weeks on a 5-point scale from 0 (never) to 4 (very often). We included subscales related to binge eating (i.e., overeating; 8 items), purging (i.e., use of diuretics, diet pills, and self-induced vomiting; 6 items), restricting (i.e., limiting food intake; 6 items), excessive exercise (i.e., I felt that I needed to exercise nearly every day; 5 items), and muscle building (i.e., use of muscle building supplements and cognitions associated with muscle building; 5 items). The EPSI has demonstrated factorial invariance across men and women, good-to-excellent internal consistency, and good 2–4-week test-retest reliability across subscales (α = 0.71–0.88; *r* = .71–0.85; Forbush et al. [17]. In this study, EPSI subscales had acceptable-to-excellent internal consistency estimates in men (αs = 0.76–0.89) and women (αs = 0.75–0.90).

### Rosenberg self-esteem scale (RSES; Rosenberg, 1979)

The RSES is a 10-item questionnaire assessing global self-esteem. Respondents indicate the extent that they agree with statements using a 4-point Likert scale ranging from 1 (strongly agree) to 4 (strongly disagree). Thus, high scores represent higher overall global self-esteem. The RSES has demonstrated good-to-excellent convergent and discriminant validity in young adults and has good 1–4-week test-retest reliability in undergraduates (*r* = .082 − .84) [31, 37, 41]. In this study, the RSES demonstrated excellent internal consistency estimates in men (α = 0.90) and women (α = 0.90).

### Statistical analyses

To address Hypothesis 1a-1b independent samples t-tests were used to examine differences in overevaluation of shape, weight and muscularity in men versus women. Hypothesis 2a-2b employed multigroup structural equation modeling involving the specification of four separate models with varying degrees of constraints. This statistical methodology allowed us to first examine structural equation models specifying overevaluation of shape-, weight-, and muscularity as predictors of ED symptoms in men and women separately (Model 1; M1). Of importance, Model 1 did not contain any restrictions and was specified by the null B rule. In other words, the model varied on 0 degrees of freedom and was unfalsifiable. The second model specified overevaluation of shape, weight, and muscularity as predictors of ED symptoms while holding regression coefficients consistent across men and women (Model 2; M2). The third model specified overevaluation of shape, weight, and muscularity as predictors of ED symptoms while holding regressions coefficients and residual variances of the ED symptom variables (each of which captures the amount of variance in each ED symptom variables that remains unexplained, following its regression on the three dependent variables) consistent across men and women (Model 3; M3). The fourth, and final model, specified overevaluation of shape, weight, and muscularity as predictors of ED symptoms while holding regression coefficients, residual variances of ED symptom variables, and pairwise covariances of the residuals of the ED symptom variables (each of which captures the amount of linear association between corresponding EPSI variables that remains unexplained, following regression on the three SAWBS variables) consistent across men and women (Model 4; M4). The specification of these models produces robust standard errors and Bentler-Sattora test statistics. These computations provide information regarding the fit of each specified model.

**Fig. 1:**
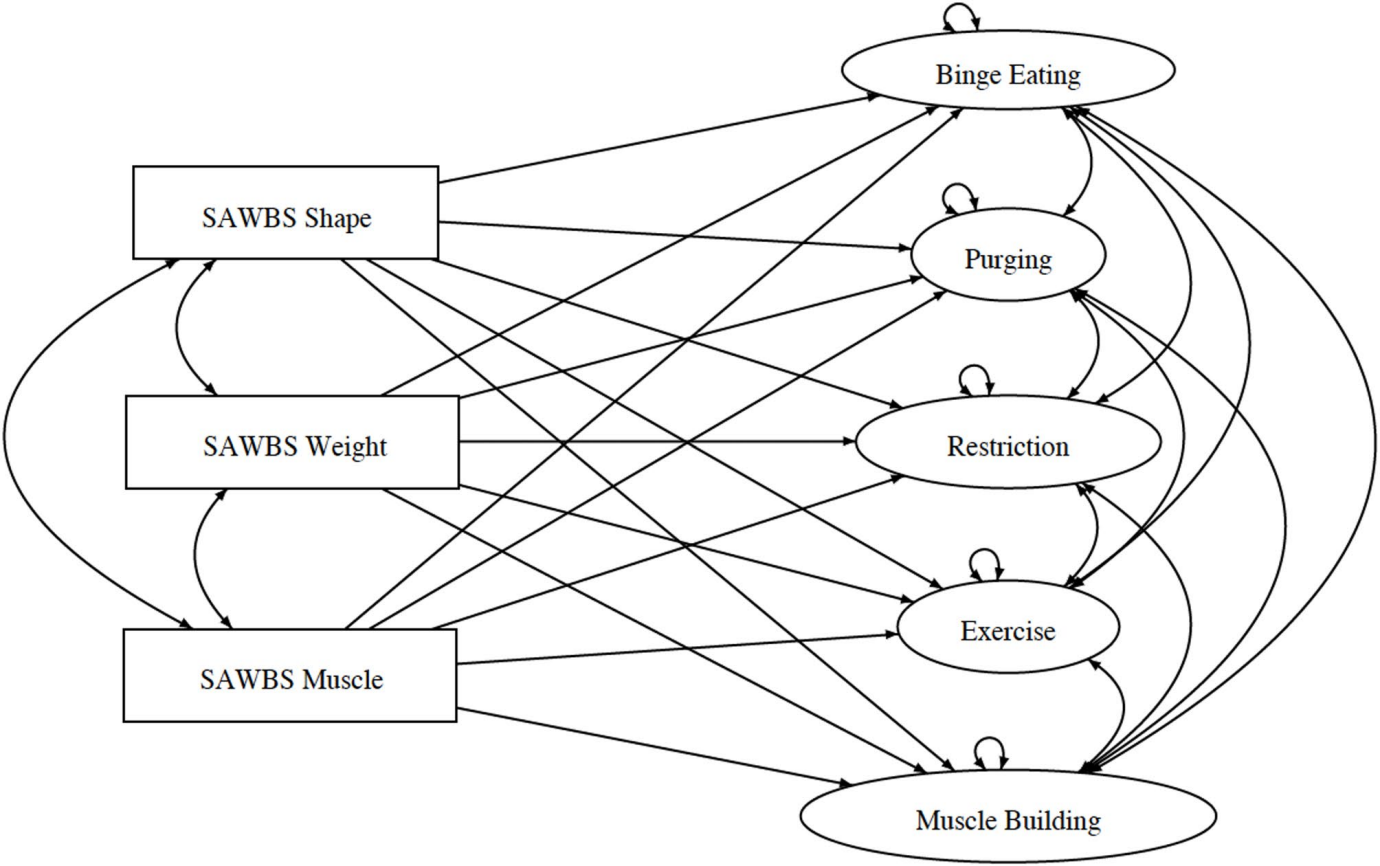
Core model depicting appearance-based self-esteem as predictors of eating disorder symptoms

Following specification of the models, a hierarchical test of the differences between Model 1 through Model 4 was conducted. These issues of invariance are addressed through the fitting of up to four multi-sample models to the data using the Lavaan package of R Lavaan [38]. All of M1 to M4 derive from what is designated, herein, as the Core Model (see Fig. 1) which was established by the Null B rule [4], and form a nested sequence of models with M1 the least, and M4 the most, restricted. The specific hypotheses expressed in 2a and 2b are assessed on the basis of estimated parameters of whichever of M1 to M4 is most consistent with the data. A variety of assumptions including assumption of normality of each variable, homogeneity of variances, and linear dependence of each variable on other variables underlie several of the statistical analyses employed. Further information concerning model specifications and assumption checking are presented in the Appendix.

### Testing procedure

Following an initial fitting of M1 to the data, the issue of Gender invariance in the regression parameters of the Core Model will be addressed by imposing the restrictions inherent to each of models M2 – M4. Decision-making regarding the suitability of these restricted models will be based on the chi-square difference testing procedure. In particular, as indicated in Table 1, the test of the hypothesis pair $\left[ {{H_0}:{\Gamma _{\left[ M \right]}}={\Gamma _{\left[ W \right]}},{H_1}:\sim {H_0}} \right]$ will be based on a chi-square difference test [M2 vs. M1 on 15 degrees of freedom]. Conditional on a retention of ${H_0}:{\Gamma _{\left[ M \right]}}={\Gamma _{\left[ W \right]}}$, the test of the hypothesis pair $\left[ {{H_0}:Diag{{\left( \Psi \right)}_{\left[ M \right]}}=Diag{{\left( \Psi \right)}_{\left[ W \right]}},{H_1}:\sim {H_0}} \right]$ will be based on a chi-square difference test of M3 versus M2 [on 5 degrees of freedom]. Finally, conditional on a retention of ${H_0}:Diag{\left( \Psi \right)_{\left[ M \right]}}=Diag{\left( \Psi \right)_{\left[ W \right]}}$, the test of the hypothesis pair $\left[ {{H_0}:{\Psi _{\left[ M \right]}}={\Psi _{\left[ W \right]}},{H_1}:\sim {H_0}} \right]$ will be based on a chi-square difference test of M4 versus M3 [on 10 degrees of freedom].

## Results

### Descriptive analyses

Descriptive information and correlations among study variables are in Table 2 (see Page 14). SAWBS Shape and Weight subscales were highly interrelated for men and women. In contrast, SAWBS Muscle had small-to-medium associations with SAWBS Shape but was unrelated to SAWBS Weight for men and women. Overall, muscle building most strongly related to overevaluation of muscularity across men and women, while purging most strongly related to overevaluation of muscularity in men. SAWBS Shape and Muscle demonstrated small-to-medium negative correlations with global self-esteem in men and SAWBS Weight and Muscle demonstrated small-to-medium negative correlations with global self-esteem in women.

Independent samples t-tests examining mean differences in ED symptoms across men and women revealed that women engaged in greater levels of purging (*t*(248) = -2.76, *p* = .003; d = -0.33) and restriction (*t*(284) = -4.55; *p* < .001; d = -0.54) than men. Alternatively, men engaged in greater excessive exercise (*t*(281) = 4.35; *p* < .001; d = 0.52) and muscle building (*t*(253) = 6.07 *p* < .001; d = 0.73) than women.

### Hypotheses 1a-1b

Findings showed that women reported higher overevaluation of shape (*t*(288) = -3.30; *p* < .001; d = -0.39) and weight (*t*(219) = -4.01; *p* < .001; d = -0.47) than men. Further, findings showed that men reported higher overevaluation of muscularity (*t*(202) = 5.44; *p* < .001; d = 0.64) than women.

### Hypotheses 2a-2b

**Fig. 2:**
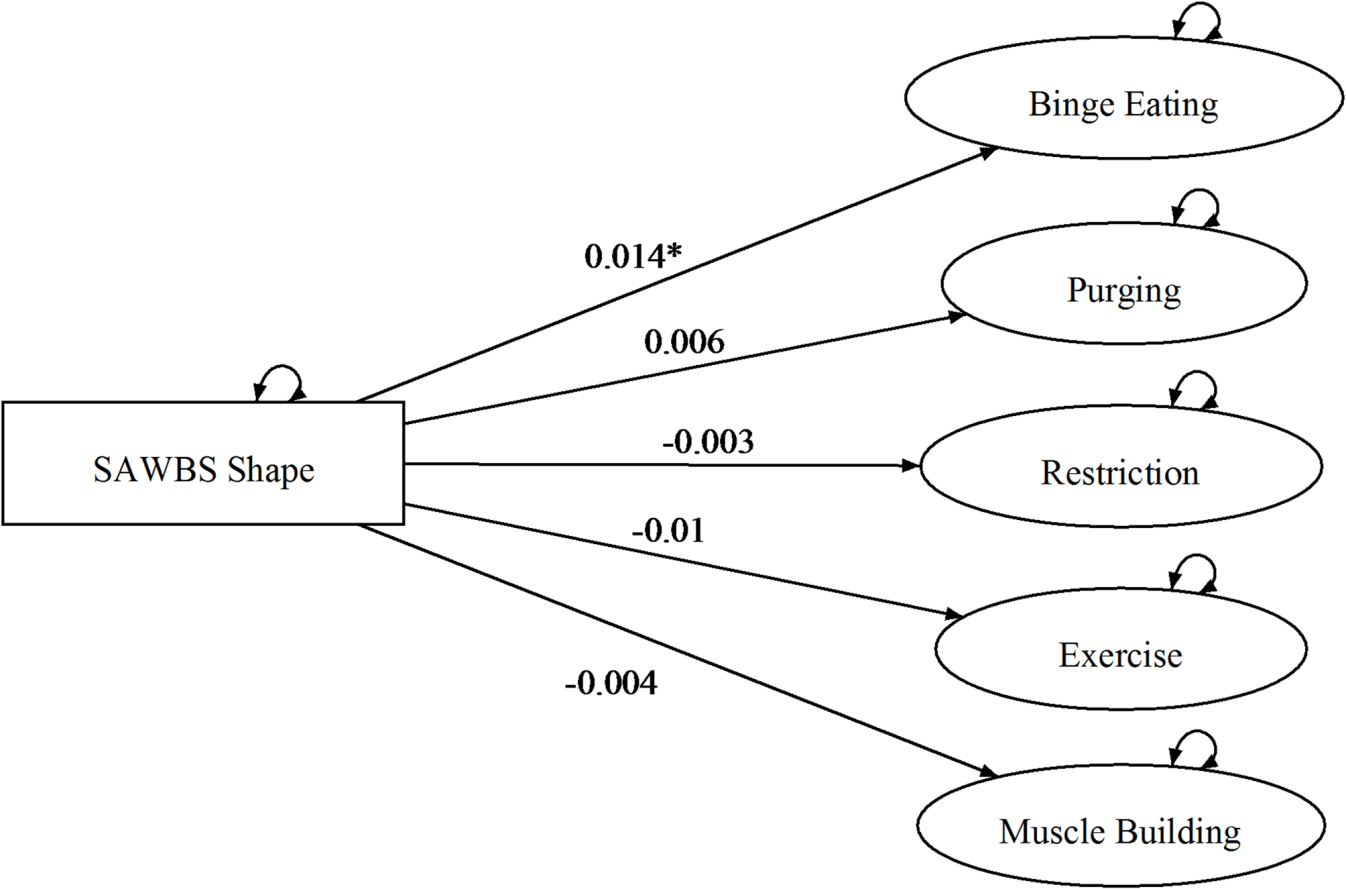
Model 2 overevaluation of shape and eating disorder symptoms across men & women. *p < .05, **p < .01, *** *p* < .001

**Fig. 3:**
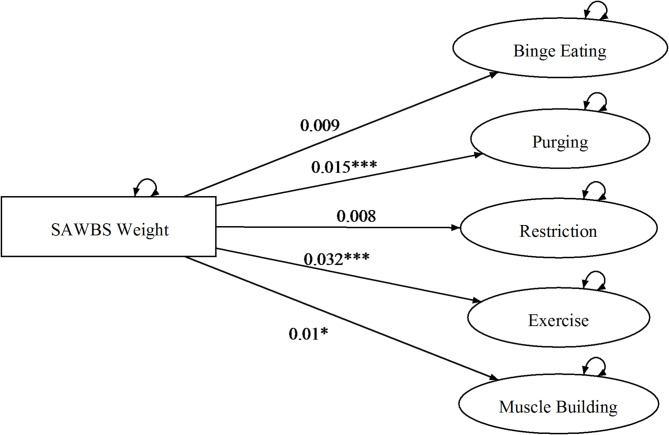
Model 2 overevaluation of weight and eating disorder symptoms across men & women. *p < .05, **p < .01, *** *p* < .001

**Fig. 4:**
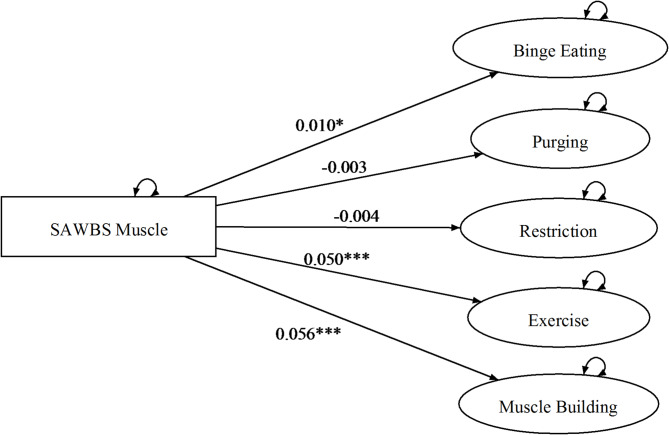
Model 2 overevaluation of muscularity and eating disorder symptoms across men & women. *p < .05. **p < .01. *** *p* < .001

The hypotheses contained within sets 2a and 2b broadly pertain to the issue of invariance, over the levels of the variable gender. The model testing procedure (see Appendix) led to the selection of M2 as the correct description of the data. Given that in M2 slope parameters are invariant over levels of Gender, this implies a rejection of the hypotheses contained in sets 2a and 2b, which posited that the pattern of relationships between the three self-esteem domains and the ED symptom variables would be different for the two genders (see Table 3). For ease of depiction, the estimated dependencies of the ED symptom variables on the three self-esteem domains are diagrammed for each facet separately in Figs. 2, 3 and 4. As can be seen, binge eating had a positive linear dependency on shape-based self-esteem; purging, excessive exercise, and muscle building had positive linear dependencies on the weight-based self-esteem; and binge eating, excessive exercise, and muscle building had positive linear dependencies on muscularity-based self-esteem.

**Table 2:** Descriptive statistics and correlations among shape, weight, and muscularity-based self-esteem, global self-esteem and disordered eating symptoms presented separately for men and women

	Self-esteem				ED symptoms					
	1. Age	2. RSES	3. Shape	4. Weight	5. Muscle	6. Binge	7. Purge	8. Restr	9. Exer	10. Muscle
1. Age	-	0.05	0.03	− 0.02	0.19*	− 0.01	− 0.05	− 0.08	− 0.10	0.00
2. RSES	0.00	-	− 0.18*	− 0.26***	0.05	− 0.34***	− 0.26**	− 0.32***	− 0.14	− 0.09
3. SAWBS Shape	0.02	− 0.23**	-	0.43***	0.19*	0.24**	0.20*	0.00	0.14	0.04
4. SAWBS Weight	− 0.07	− 0.11	0.37***	-	− 0.07	0.20*	0.40***	0.10	0.30***	0.12
5. SAWBS Muscle	− 0.08	− 0.26**	0.28***	0.07	-	0.06	0.11	0.01	0.25**	0.40***
6. EPSI Binge	0.10	− 0.38***	0.15	0.10	0.20*	-	0.46***	0.18*	0.34***	0.30**
7. EPSI Purge	0.05	− 0.24**	0.24**	0.24**	0.01	0.23**	-	0.22**	0.27**	0.17*
8. EPSI Restrict	− 0.10	− 0.10	0.03	0.04	− 0.06	− 0.07	0.27**	-	0.16*	0.15
9. EPSI Exercise	− 0.14	0.05	0.12	0.16	0.43***	0.15	0.12	0.01	-	0.60***
10. EPSI Muscle	− 0.05	− 0.15	0.23	0.10	0.54***	0.23**	0.17	0.14	0.71***	-
Means (SD)										
Men	19.33 (1.61)	27.76 (6.58)	6.74 (7.51)	4.74 (5.80)	7.82 (10.66)	9.25 (5.53)	0.73 (2.10)	6.39 (5.10)	9.67 (5.65)	6.74 (5.26)
Women	19.10 (1.47)	26.84 (5.84)	9.89 (8.78)	8.91 (11.14)	2.48 (4.99)	10.50 (6.84)	1.62 (3.20)	9.35 (5.90)	6.76 (5.61)	3.38 (3.93)

*SAWBS* = Shape and Weight Based Self-Esteem Inventory, *EPSI* = Eating Pathology Symptoms Inventory. Correlations for men are below the diagonal. Correlations for women are above the diagonal. Raw values are presented for descriptive information. *p < .05, **p < .01, *** *p* < .001

**Table 3:** Comparison of fit of model 1 through model 3 examining gender differences in the relationship between overevaluation of shape, weight, and muscularity and eating disorder symptoms

	Number of input points	Parameters estimated	df	$\:\chi\:$2	*p*	df_diff_	$\:\chi\:$ ^2^_diff_	*p*_diff_
M1	72	72	0	0	0.00	-	-	
M2	72	57	15	10.72	0.69	15	10.17	0.81
M3*	72	52	20	25.80	0.07	5	15.33	0.008

M1 = Model 1, M2 = Model 2, M3 = Model 3; *rejected null hypothesis

## Discussion

The Transdiagnostic Model of Eating Disorders posits that overevaluating one’s self-esteem on shape and weight is a central factor in the development and maintenance of ED symptoms. However, there exist crucial differences in how men and women evaluate their bodies, which may result in gender differences in the aspects of appearance that they value in deriving their self-esteem. Yet, no research has sought to examine whether overevaluation of muscularity, in addition to overevaluation of shape and weight, may be associated with ED symptoms in men and women. Thus, this study first examined whether men and women differed in the extent to which they valued shape, weight, and muscularity in their self-esteem. Women reported greater overevaluation of shape and weight than men, while men endorsed greater overevaluation of muscularity than women. Second, we examined whether associations between overevaluation and ED symptoms varied by gender. Overall, relationships that emerged between overevaluation of shape, weight, and muscularity and ED symptoms did not vary by gender. Altogether, results provide nuanced information regarding the importance of examining muscularity, alongside shape and weight, when examining how overevaluation of appearance relates to ED symptoms in men and women.

Consistent with hypotheses, women endorsed overevaluation of shape and weight more often than men and men endorsed overevaluation of muscularity more often than women. However, it is worth noting that overall levels of basing self-esteem on shape, weight or muscularity in the present sample were low; a result which may be reflective of the university sample. Research with female university students, women “at-risk” of developing an eating disorder, and clinical samples of women with eating disorders (e.g., anorexia nervosa, bulimia nervosa) demonstrates that basing self-esteem on appearance relates to decreased overall self-esteem [19, 42, 43]; yet this study is among the first to clarify how overevaluation of different forms of appearance relate to self-esteem in men. Further, past research highlights muscularity concerns prominence for men [18, 34], yet no research has investigated the relevance of muscularity as a construct distinct from shape and weight. Thus, this finding extends past research and highlights that overevaluation of muscularity may be important to assess alongside shape and weight across genders.

While important differences in the forms of appearance which men and women based their self-esteem, the associations between overevaluation of shape, weight and muscularity and ED symptoms did not vary between men and women. These findings suggest that although men are more likely to over evaluate muscularity than women, and women are more likely to over evaluate shape or weight than men, the presence of overevaluation of any form of appearance may be associated with ED symptoms. Thus, while men and women might internalize different body ideals and engage in different ED behaviours, the presence of any form of overevaluation may suggest risk for ED symptoms in men and women. These findings underscore the relevance of evaluation of muscularity for men and women. Indeed, research suggests that women internalize thin and muscular ideals concurrently resulting in internalization of a ‘fit-ideal’ [44]. Internalization of the fit-ideal relates to pathological exercise behaviours in women [11] and internalization of the muscularity-ideal relates to pathological exercise in men [15]. Thus, this research is consistent with these findings, and suggests that weight and muscularity may be appearance categories that, when overvalued, relate to excessive exercise and muscle building in men and women. It may be that the popularization of the ‘fit-ideal’ for women has increased the importance of muscularity for women, resulting in the relevance of muscularity-based self-esteem for both men and women. These findings underscore the evolving nature of body ideals across genders and may provide evidence of the importance of targeting muscular and thin ideals in existing treatments across genders.

### Strengths, limitations, and future directions

This is the first study to include muscularity as a distinct appearance-based evaluative category separate from shape and weight when conceptualizing overevaluation of appearance, despite research showing that men and women prioritize different ideals which relates to different ED symptoms [26, 27, 29]. Thus, the assessment of the degree that men and women base their self-esteem on muscularity as a domain distinct from other appearance-based categories such as shape and weight. Furthermore, we translated the SAWBS [19], a paper and pencil measure of overevaluation of shape and weight, into an easily used online measure. The online measure automatically calculates the degrees that each domain “makes up” the pie, omitting the need for researchers to measure and document participants’ physical drawings. Last, we recruited a diverse sample of men and women. Over 50% of the sample identified as South Asian, East Asian, or Southeast Asian. Of importance, South Asian women are particularly at risk for ED symptoms (see for review, Khasru [28], and more research needs to be done to understand risk factors for ED symptoms in diverse samples [9].

Findings must be interpreted within the context of limitations. First, the study used a cross-sectional design, limiting the ability to make causal claims and determine directionality between overevaluation and ED symptoms. It may be that there is a bidirectional relationship between the presence of ED symptoms and overevaluation, wherein overevaluation increases in response to ED symptoms which also may maintain these symptoms. Understanding not only temporality, but the direction of the relationships between overevaluation of muscularity and ED symptoms may be an important next step in understanding the role of overevaluation of muscularity and how it may function similarly or differently to overevaluation of shape and weight. Further, despite recruitment of a large and diverse sample with a nearly equal gender distribution, we only analyzed cisgender individuals. Non-binary and transgender young adults are at particularly high-risk for the development of ED symptoms [2, 7]. Thus, future research should examine how appearance-based self-esteem manifests and relates to ED symptoms in gender diverse populations. Finally, the present study ascribed pre-defined categories of “shape”, “weight”, and “muscularity” which may have biased responses. For instance, shape which was defined as “the way your clothes fit” may encompass overevaluation of muscularity, such as a focus on the way clothes fit around or are enhanced by muscle’s size or tone. Thus, qualitative research examining the forms of muscularity and muscle ideals most relevant to men and women, whether they be labelled as shape, weight or muscularity overevaluation, may be needed to understand how qualitative differences in appearance-based self-esteem more specifically relate to ED symptoms. A better understanding of appearance-based self-esteem can aid in tailoring the transdiagnostic model and existing treatments and assessment.

## Supplementary Information

Supplementary material 1.

## Data Availability

Data is available upon request.
