# Supplementary material for: How do I evaluate myself? The importance of examining overevaluation of muscularity in risk for eating disorder symptoms
Source: J Eat Disord. 2025 Oct 23;13:233. doi: 10.1186/s40337-025-01419-3 (PMC12551218; doi:10.1186/s40337-025-01419-3)
Supplement: Supplementary file 1 — Supplementary material 1. [file 40337_2025_1419_MOESM1_ESM.docx]

**Appendix**

**Table A**

*Model 1, 2, 3, and 4’s Multigroup Structural Equation Modeling Specifications*

|  | Restrictions | Number of Input Points | Parameters Estimated | df | df_diff_ |
| --- | --- | --- | --- | --- | --- |
| M1 | - | 72 | 72 | 0 |  |
| M2 | $\left[H_{0}:\Gamma_{\left[M\right]}=\Gamma_{\left[W\right]},H_{1}:\sim H_{0}\right]$ | 72 | 57 | 15 | 15 |
| M3 | $\left[H_{0}:Diag\left(\Psi\right)_{\left[M\right]}=Diag\left(\Psi\right)_{\left[W\right]},H_{1}:\sim H_{0}\right]$ | 72 | 52 | 20 | 5 |
| M4 | $\left[H_{0}:\Psi_{\left[M\right]}=\Psi_{\left[W\right]},H_{1}:\sim H_{0}\right]$ | 72 | 42 | 30 | 10 |

Note. M1 = Model 1, M2 = Model 2, M3 = Model 3, M4 = Model 4

Assumptions regarding the normality of the distribution of the dependent variables within the population were examined through visual inspection of a Q-Q plot and confidence intervals for skewness and kurtosis. Homogeneity of variances was examined through Levene’s test which produces an inferential statistic to assess the equality of variances of variables for two groups. Lastly, linear dependence of the dependent variables and independent variables were examined using pairwise scatterplots with a projected LOESS estimated curve of best fit.

***Assumption of Normality.*** The assumption of normality of each variable used in analyses was examined using a Q-Q plot and confidence intervals for skewness and kurtosis. Mild evidence of non-normality of dependent and independent variables were observed. However, independent samples t-tests are robust against non-normality and thus, no transformations were performed.

***Assumption of Homogeneity of Variances***. For each of the independent sample’s t-tests, the assumption of homogeneity of variances was examined using Levene’s test with alpha set to .01. For the variables SAWBS Weight, SAWBS Muscle, EPSI Binge, EPSI Purge, and EPSI Muscle Building, Levene’s indicated a violation of the assumption of homogeneity of variances. Thus, we employed the Welch-Satterthwaite alternative for the tests involving variables that violated assumptions of homogeneity of variance (i.e., SAWBS Weight, SAWBS Muscle, EPSI Binge, EPSI Purge, and EPSI Muscle Building). The standard independent samples t-test was used for those variables that did not violate this assumption.

***Assumption of Linearity.*** We estimated correlations to capture the degree of association between pairs of variables. In particular, they stand as input into hypotheses 2a and 2b and Models M1 to M4, which are estimated as the basis for addressing hypotheses 2a and 2b. Employing correlations when a pairwise relationship is, in fact, nonlinear can result in misspecification errors in the fitting of models and, more generally, incorrect empirical claims. The issue of linearity was checked using pairwise scatterplots and, for each, a LOESS estimated curve of best fit was projected onto scatterplots of EPSI variables on SAWBS variables, EPSI variables on EPSI variables, SAWBS variables on SAWBS variables, and RSES on SAWBS variables. Linearity characterized the relationship of the majority of EPSI variables on SAWBS variables, EPSI variables on EPSI variables, SAWBS variables on SAWBS variables, and RSES on SAWBS variables. Exceptions were EPSI Purge on SAWBS variables, EPSI Binge on SAWBS Shape and Weight, and SAWBS Weight on SAWBS Muscle. For those in violation of the assumption, the nonlinearity in evidence was deemed modest and accordingly, we concluded that a linear approximation was an acceptable platform on which to found the analyses undertaken.
